# Innate lymphoid cells as central regulators of tissue homeostasis: implications for allergic diseases

**DOI:** 10.3389/falgy.2026.1954214

**Published:** 2026-10-19

**Authors:** Kapil Sirohi, Sangya Yadav

**Affiliations:** 1 Division of Allergy and Immunology, Department of Medicine, National Jewish Health, Denver, CO, United States; 2 Department of Medicine, University of Colorado, Aurora, CO, United States

**Keywords:** allergy and immunology disorders, asthma, group 2 innate lymphoid cell (ILC2), innate lymphoid cells (ILC), tissue homeostasis

## Abstract

Barrier tissues continuously encounter environmental challenges while maintaining immune tolerance and tissue integrity. Preservation of homeostasis within the skin, respiratory tract, and gastrointestinal mucosa requires coordinated communication among epithelial, stromal, neuronal, vascular, and immune cells. Innate lymphoid cells (ILCs) have emerged as key regulators of these processes owing to their strategic localization within barrier tissues and their capacity to rapidly integrate tissue-derived signals. Although initially classified as innate counterparts of helper T-cell subsets, accumulating evidence demonstrates that mature ILC identity is continuously shaped by developmental programming, tissue-specific environmental cues, and functional plasticity. Beyond their established roles in host defense, ILCs regulate epithelial maintenance, tissue repair, extracellular matrix remodeling, neuroimmune communication, and resolution of inflammation through extensive interactions with structural and immune cells. Under physiological conditions, these activities preserve barrier integrity and restore tissue homeostasis following injury. However, persistent epithelial damage and chronic exposure to environmental stimuli disrupt these regulatory networks, resulting in sustained ILC activation, impaired resolution, pathological remodeling, and progressive organ dysfunction. With particular emphasis on ILC2s in allergic disease, this review summarizes recent advances in ILC development, tissue adaptation, and multicellular communication, and examines how disruption of these homeostatic programs contributes to asthma, chronic rhinosinusitis with nasal polyps, eosinophilic gastrointestinal diseases, and atopic dermatitis. Finally, we highlight emerging technologies and future directions that may facilitate restoration of tissue homeostasis as a therapeutic strategy for chronic allergic diseases.

## 1 Introduction

Barrier tissues, including the skin, respiratory tract, and gastrointestinal mucosa, represent the primary interface between the host and the external environment. These tissues are continuously exposed to allergens, pathogens, commensal microorganisms, pollutants, and mechanical injury, necessitating rapid yet tightly regulated immune responses that eliminate threats while preserving tissue integrity. Successful immunity at these sites depends not only on pathogen clearance but also on the timely restoration of tissue homeostasis through coordinated interactions among structural cells, resident immune populations, and the local microenvironment. Failure of these regulatory mechanisms results in persistent inflammation, aberrant tissue remodeling, and progressive loss of barrier function, pathological features that characterize many chronic allergic diseases (1, 2).

Among the immune populations residing within barrier tissues, innate lymphoid cells (ILCs) have emerged as important regulators of both immune responses and tissue homeostasis. Unlike adaptive lymphocytes, ILCs lack somatically rearranged antigen receptors but rapidly respond to tissue-derived cytokines, neuronal mediators, metabolites, and other environmental signals. Their strategic localization within barrier organs enables them to continuously monitor changes in the local microenvironment and coordinate early immune responses while preserving epithelial integrity, facilitating tissue repair, and promoting restoration of tissue homeostasis before adaptive immunity is fully engaged. Consequently, ILCs are increasingly recognized not only as rapid cytokine producers but also as integral components of tissue-resident regulatory networks that maintain organ function throughout life (1, 3, 4).

The modern ILC field emerged from discoveries spanning nearly two decades. Identification of lymphoid tissue inducer (LTi) cells established the first member of this lymphocyte family and revealed an essential role for innate lymphocytes in secondary lymphoid organogenesis (5). The field expanded rapidly following the independent reports by Neill and colleagues, Moro and colleagues, and Price and colleagues in 2010, which identified innate cells capable of producing type 2 cytokines in response to epithelial-derived alarmins, establishing what are now recognized as group 2 innate lymphoid cells (ILC2s) (6–8). Together with the subsequent identification of additional ILC subsets and the unified classification proposed by Spits and colleagues, these discoveries established ILCs as a distinct lineage of innate lymphocytes and transformed our understanding of tissue immunity (9).

Initially, ILCs were viewed primarily as innate counterparts of CD4⁺ helper T-cell subsets because of their shared transcription factors and cytokine profiles. Although this paradigm remains valuable for classification, accumulating evidence demonstrates that mature ILC identity is shaped not only by developmental programming but also by continuous interactions with the surrounding tissue environment. Advances in single-cell transcriptomics, spatial biology, lineage-tracing approaches, and high-dimensional imaging have revealed remarkable tissue-specific specialization among resident ILC populations. Local cytokines, stromal and epithelial interactions, neuronal inputs, microbial metabolites, and metabolic signals collectively influence ILC phenotype, functional plasticity, and long-term maintenance, enabling these cells to adapt to the physiological demands of individual organs while preserving a common developmental framework (10–12).

This evolving understanding has expanded the functional framework of ILC biology. In addition to their well-established roles in coordinating rapid immune responses, ILCs contribute to epithelial maintenance, tissue repair, extracellular matrix remodeling, neuroimmune communication, metabolic adaptation, vascular integrity, and the resolution of inflammation through continuous crosstalk with epithelial, stromal, neuronal, and adaptive immune cells. Rather than representing separate biological activities, immune defense and tissue homeostasis are closely integrated functions that enable ILCs to respond effectively to environmental challenges while preserving tissue integrity and organ function (2, 4).

However, the same regulatory pathways that protect barrier tissues can become maladaptive when chronically activated or inadequately controlled. Persistent exposure to allergens, repeated epithelial injury, microbial dysbiosis, or alterations in the tissue microenvironment disrupt the tightly regulated circuits governing ILC activation, leading to sustained cytokine production, impaired resolution of inflammation, pathological remodeling, and fibrosis. Increasing evidence indicates that dysregulated ILC responses contribute to the pathogenesis of asthma, chronic rhinosinusitis with nasal polyps, atopic dermatitis, eosinophilic gastrointestinal diseases, and other allergic disorders. Accordingly, allergic diseases can be viewed as conditions in which dysregulated immune responses and impaired tissue homeostasis reinforce one another, with ILCs contributing to both protective immunity and pathological remodeling depending on the tissue context (2, 13).

Although the broader ILC family provides the developmental and tissue-level framework for this review, ILC2s are particularly relevant to allergic disease because they reside at epithelial barriers and respond rapidly to interleukin (IL)-25, IL-33, thymic stromal lymphopoietin (TSLP), and local neuronal and metabolic signals. Accordingly, we use “ILCs” to denote functions shared across subsets or involving multiple populations, and “ILC2s” for subset-specific mechanisms implicated in type 2 inflammation. The following discussion of ILC development, tissue residency, and plasticity provides the context for understanding how tissue-adapted ILC2 responses can shift from protective repair to persistent inflammation when epithelial injury fails to resolve (2, 4, 14).

## 2 Establishing innate lymphoid cell identity: developmental programming, tissue adaptation, and functional plasticity

The identification of innate lymphoid cells (ILCs) transformed our understanding of innate immunity by revealing a family of lymphocytes that lack somatically rearranged antigen receptors yet rapidly coordinate immune responses and tissue adaptation. Although ILCs are classified into distinct subsets according to developmental origin, transcription factor expression, and effector function, mature ILC identity is not established by lineage commitment alone. Developmental programming provides the molecular framework for lineage specification, whereas signals encountered during and after tissue colonization shape organ-specific phenotypes and functional specialization. Throughout adult life, mature ILCs retain the capacity to adjust their transcriptional and functional programs in response to changes in the local microenvironment. Developmental programming, tissue education, and functional plasticity therefore act together to generate the diversity of ILC populations found across barrier tissues and provide the biological foundation for their roles in immune defense and tissue homeostasis (1, 12, 15).

### 2.1 Developmental programming establishes functional potential

ILCs arise through lymphoid developmental pathways that overlap initially with those generating B cells, T cells, and natural killer (NK) cells before diverging toward distinct innate lymphoid fates. In adult mice, much of this process occurs within the bone marrow, whereas embryonic and fetal hematopoietic sites contribute substantially to the initial establishment of tissue ILC compartments. Unlike adaptive lymphocytes, whose functional diversity depends on somatic antigen-receptor rearrangement and clonal selection, ILCs acquire lineage-associated transcriptional and epigenetic programs during development that permit rapid, antigen-independent responses following infection or tissue injury (1, 16).

Commitment to the ILC lineage is controlled by an interconnected network of transcription factors that restrict alternative lymphocyte fates while establishing innate lymphoid competence. Early regulators including nuclear factor interleukin-3-regulated protein (NFIL3), thymocyte selection-associated high mobility group box protein (TOX), T-cell factor 1 (TCF-1), GATA3, and inhibitor of DNA binding 2 (Id2) act through overlapping and context-dependent functions rather than as a simple linear cascade. Id2 suppresses E-protein activity and thereby limits B- and T-cell differentiation while supporting innate lymphoid lineage commitment (5). NFIL3 and TOX contribute to the generation of multipotent ILC progenitors, whereas TCF-1 and GATA3 support subsequent lineage specification and maturation (17–20). The timing, magnitude, and combination of these transcriptional inputs collectively determine developmental competence.

Identification of intermediate progenitor populations further refined the model of ILC ontogeny. Klose and colleagues described the common helper innate lymphoid progenitor (CHILP), which generates helper-like ILC1s, ILC2s, ILC3s, and lymphoid tissue inducer cells while lacking conventional B-, T-, and NK-cell potential (21). Constantinides and colleagues subsequently identified promyelocytic leukemia zinc finger (PLZF)-expressing ILC progenitors capable of producing ILC1s, ILC2s, and ILC3s but with limited capacity to generate conventional lymphoid tissue inducer cells (22). Together with the identification of additional intermediate populations, these studies established that ILC differentiation proceeds through progressively restricted developmental states rather than through a single lineage decision (18, 23). For ILC2s specifically, murine genetic studies identify Bcl11b as a requirement for lineage development, linking the general progenitor framework to the type 2 effector compartment relevant to allergy (23, 24).

Recent findings have complicated the traditional view that all ILC development follows a fetal-liver-to-adult-bone-marrow sequence. In the human embryo, Ni and colleagues identified yolk-sac-derived lymphoid-biased progenitors before the appearance of definitive hematopoietic stem cells. These progenitors showed preferential potential to generate ILCs and dendritic cells and displayed migratory relationships with early fetal-liver populations, indicating that components of the human innate lymphoid system are established through hematopoietic-stem-cell-independent developmental waves (25). In mice, Wang and colleagues demonstrated that fetal-liver-derived ILC progenitors seed developing organs, where local niches direct their differentiation into tissue-associated precursor populations that can persist into adulthood and contribute to local ILC maintenance independently of continuous bone-marrow output (26). These observations support a layered model of ILC ontogeny in which embryonic, fetal, and adult hematopoietic sources contribute differently according to developmental stage, tissue, and inflammatory context.

As differentiation proceeds, lineage-associated transcription factors establish the principal functional programs of mature ILC subsets. T-bet supports ILC1 differentiation and interferon-*γ* production (27), GATA3 is required for ILC2 development and maintenance (28, 29), and ROR*γ*t specifies ILC3 and lymphoid tissue inducer cell identity while regulating IL-17- and IL-22-associated functions (30, 31). These factors should not, however, be interpreted as permanently fixed lineage labels. T-bet can be induced in subsets of mature ILC2s and ILC3s under inflammatory conditions, whereas reduced ROR*γ*t expression accompanies the acquisition of ILC1-like features by some ILC3s (15, 32). Lineage-associated transcription factors therefore establish developmental potential and functional bias while leaving mature cells responsive to environmental reprogramming. Human ILC2 experiments also establish a requirement for GATA3 in cytokine production, providing functional support beyond the murine developmental evidence (33).

Epigenetic organization provides an additional level of control over this developmental potential. Lineage-associated enhancers and accessible chromatin regions establish the regulatory architecture required for rapid cytokine production, but mature ILC populations also retain accessible regulatory elements associated with alternative functional programs. Single-cell analysis of human peripheral-blood ILCs has revealed substantial heterogeneity in both transcriptional profiles and chromatin accessibility, including distinct ILC2 and ILC progenitor states with differentially poised regulatory regions (34). These findings help explain how ILCs maintain recognizable lineage identities while remaining capable of functional adaptation.

Human studies broadly support the developmental framework established in mice while revealing important differences in progenitor composition, tissue distribution, and surface phenotype. Circulating human ILC progenitors capable of generating multiple mature ILC subsets have been identified in peripheral blood and secondary lymphoid tissues (35). Following migration to peripheral organs, these progenitors can complete their maturation under the influence of local environmental signals, indicating that developmental programming establishes lineage competence while mature tissue identity continues to develop after tissue entry.

Translational interpretation requires care. Murine parabiosis and fate-mapping experiments establish local persistence or lineage relationships under defined experimental conditions, but human biopsy and single-cell studies typically yield cross-sectional associations rather than direct measurements of cell turnover or fate. Human circulating ILC progenitors further complicate extrapolation from mouse tissue-residency estimates. Surface markers, including chemoattractant receptor-homologous molecule expressed on T helper 2 cells (CRTH2) and cytokine receptors, can change with activation and tissue processing, so marker-defined human subsets may not map one-to-one onto murine populations. Species- and tissue-dependent differences in receptor expression and signaling should therefore be tested rather than assumed; concordant transcriptional profiles alone do not demonstrate equivalent functional behavior or causal roles in disease (3, 35, 36).

Human ILC populations also display phenotypic diversity that extends beyond the canonical ILC1, ILC2, and ILC3 classification. Liu et al. identified conventional CRTH2 + interleukin-7 receptor *α* (IL-7R*α*)+ ILC2s together with CRTH2-IL-7R*α*- populations capable of producing type 2 cytokines in response to epithelial alarmins (37). These observations demonstrate that classification based exclusively on a limited set of surface markers can underestimate human ILC diversity. Developmental history, activation state, tissue localization, and environmental adaptation must therefore be considered alongside canonical immunophenotypic definitions.

### 2.2 Tissue residency and environmental education define mature ILC identity

Developmental programming establishes the capacity of ILCs to acquire specialized functions, but it does not fully determine the phenotype of mature cells. Following migration into peripheral tissues, ILCs undergo continuing adaptation through interactions with the local microenvironment. Signals from epithelial cells, fibroblasts, endothelial cells, neurons, resident immune populations, microbial communities, and tissue metabolites shape organ-specific transcriptional programs that allow ILCs to respond to the physiological requirements of individual tissues. Mature ILC identity is therefore established jointly by developmental origin and continued environmental education within spatially organized tissue niches (3, 11, 12).

Most mature ILC populations are maintained predominantly within tissues. Parabiosis studies demonstrated that ILCs in the lung, intestine, adipose tissue, skin, and other organs persist mainly through local maintenance and self-renewal rather than continuous replacement from the circulation (3). Long-term residency enables these cells to integrate information concerning epithelial integrity, stromal activity, vascular function, neuronal signaling, nutrient availability, and microbial composition before initiating immune responses. This close association with the tissue environment allows resident ILCs to respond rapidly to local perturbations while maintaining functions adapted to the organ in which they reside.

Tissue residency does not mean that ILC compartments are developmentally closed. Circulating progenitors and activated mature ILCs can enter peripheral tissues during development, inflammation, or injury, contribute to local responses, and acquire features of resident populations (12, 35). The relative contribution of local persistence and recruited cells differs among tissues and physiological conditions. Tissue ILC pools are therefore best viewed as relatively stable communities sustained primarily by resident cells while retaining the capacity for selective recruitment and local differentiation.

Single-cell studies have shown an association between anatomical location and mature ILC identity. Gury-BenAri and colleagues identified extensive transcriptional heterogeneity among intestinal ILCs, including intermediate cellular states that were not adequately represented by the classical ILC1, ILC2, and ILC3 framework (11). Ricardo-Gonzalez and colleagues showed that ILC2s isolated from different organs retain a conserved lineage-associated transcriptional program while acquiring distinct tissue-specific signatures reflecting their local environments (12). More recent integration of human single-cell datasets across blood, lymphoid tissue, healthy mucosa, inflamed mucosa, and tumors similarly identified both conserved lineage features and strong tissue-specific imprinting within human ILC populations (38). Single-cell transcriptomic analysis of human bone marrow, spleen, and abdominal and thoracic lymph nodes has further shown that ILC composition and maturation states differ between anatomical sites, reinforcing the view that conventional subset labels encompass multiple tissue-conditioned cellular states (39).

Tissue education occurs within multicellular niches rather than through exposure to isolated soluble mediators. Epithelial alarmins can activate ILC2s, while stromal and myeloid populations provide survival, positioning, and regulatory signals (4, 40). These interactions are not uniformly activating: the outcome depends on the ILC subset present, the tissue and inflammatory context, and whether the signal is transient or sustained. Evidence for a specific niche interaction is strongest when spatial observations are combined with perturbation; co-localization or ligand–receptor inference alone cannot establish that the proposed interaction occurs *in vivo* (39, 40).

Human spatial mapping places ILC subsets non-randomly within lymphoid tissues and identifies proximity to corresponding T-cell populations (39). This adds anatomical information that dissociated single-cell data cannot provide, but the rarity of ILCs, restricted marker panels, and cross-sectional sampling limit the identification of stable interactions and the direction of signaling. Thus, spatial and transcriptional studies support a model of tissue-conditioned ILC identity, not a complete causal map of human barrier-tissue niches (38, 39).

Neuronal and metabolic signals provide additional layers of environmental instruction. ILC2s express receptors for multiple neuropeptides and neurotransmitters, allowing direct communication with peripheral neuronal circuits. Neuromedin U promotes ILC2 activation during helminth infection and allergic inflammation, whereas β_2_-adrenergic signaling restrains excessive type 2 immune responses (14, 41). Nutrient availability, oxygen tension, lipid mediators, vitamins, and microbiota-derived metabolites also influence ILC survival and function. Dietary metabolites regulate intestinal ILC3 activity through the aryl hydrocarbon receptor, while lipid mediators can either enhance or inhibit ILC2 responses depending on the surrounding inflammatory environment (42, 43).

Tissue education is an ongoing process rather than a single event at organ entry. Cross-organ ILC2 profiles and intestinal single-cell analyses support a combination of conserved lineage features and local environmental imprinting (11, 12). However, differences between organs may also reflect unequal activation, cell recruitment, and sampling. The stability of these signatures during disease resolution remains to be determined.

An important distinction is that tissue-specific transcriptional differences do not, by themselves, demonstrate irreversible reprogramming. The intestinal profiling studies resolve heterogeneous cell states, whereas parabiosis tests exchange between compartments; neither approach alone establishes the molecular signal responsible for a given state (3, 11). The inference is strongest when anatomical mapping is combined with perturbation of candidate niche signals, as in experimental stromal–ILC niche studies (40, 44).

Taken together, mouse parabiosis supports predominantly local ILC maintenance in the tissues tested, whereas human circulating progenitors demonstrate that tissue compartments need not be closed (3, 35). These findings are complementary, not directly conflicting measurements of the same process. Likewise, spatial proximity identifies potential cellular partners but does not prove direct signaling. Longitudinal human sampling and functional perturbation are needed to distinguish durable tissue imprinting from transient activation (11, 39).

### 2.3 Functional plasticity and adaptive heterogeneity of innate lymphoid cells

ILC plasticity encompasses changes in cytokine output, receptor expression, and transcriptional state, but these phenomena should not all be equated with lineage conversion. Human cytokine-culture experiments demonstrate that ILC2s can acquire ILC1-like properties, whereas mouse fate-mapping and transcription-factor studies examine developmental history under defined conditions (45, 46). The central question is which changes persist after the inflammatory stimulus ends and whether they occur at a meaningful frequency in human allergic tissue.

Evidence for ILC plasticity first emerged from studies showing that inflammatory cytokines can reversibly alter the phenotype of mature ILC subsets. Human ILC2s exposed to IL-12 and IL-1β upregulate T-bet, gain responsiveness to IL-18, and produce interferon-γ while reducing canonical type 2 functions, thereby acquiring ILC1-like characteristics (45, 47, 48). IL-4 can promote recovery of ILC2-associated features in human culture systems (47). These observations indicate that environmental cytokines can redirect ILC2 effector function without necessarily erasing developmental history. Silver et al. linked ILC1-like shifts to infection or smoke exposure in lung models and to COPD severity in patients. This provides an airway example of plasticity, but COPD evidence does not by itself establish the same process in allergic asthma (48).

Comparable flexibility occurs within the ILC3 compartment. Exposure to IL-12, IL-15, and related inflammatory mediators can promote reduced ROR*γ*t expression, induction of T-bet, and acquisition of interferon-γ-producing ILC1-like phenotypes (32, 46, 49). In human intestinal cultures, IL-1β and IL-23 can support the reacquisition of ILC3-associated characteristics, demonstrating that the direction of plasticity depends on the cytokine environment (49). The balance between ILC3 and ILC1-like states is controlled not only by inflammatory cytokines but also by transcriptional stabilizers, microbial signals, dietary factors, and circadian pathways. Recent work showing that REV-ERB*α* and REV-ERBβ help preserve intestinal ILC3 identity further illustrates that functional plasticity is constrained by tissue-specific regulatory circuits rather than occurring randomly (50).

The evidence therefore supports environmentally responsive ILC programs rather than unrestricted lineage instability. Inflammatory cytokines can redirect effector function, while tissue-specific regulatory circuits can preserve identity. These observations stem from different experimental settings and need not be contradictory (32, 45, 50). The physiological significance of a transitional state depends on its persistence, location, and functional output, not merely on co-expression of lineage markers.

Functional heterogeneity also reflects processes other than short-term plasticity. Long-term residence within individual organs generates relatively stable transcriptional and epigenetic programs that distinguish pulmonary, intestinal, cutaneous, adipose, and lymphoid-tissue ILC populations despite shared developmental origins. Single-cell RNA sequencing and chromatin-accessibility analyses have shown that even phenotypically similar human ILCs contain multiple subpopulations with distinct activation states and regulatory potential (34, 38, 39). Some of this diversity reflects developmental history, some reflects tissue adaptation, and some reflects current activation. Surface phenotype alone therefore cannot reliably distinguish stable subsets from transient functional states.

Human ILC classification is particularly sensitive to tissue access, dissociation, activation-induced changes in surface markers, and the limited number of markers used in some studies to identify these populations. Accordingly, estimates of subset abundance or apparent conversion may differ without implying genuine context or the underlying biological process (34, 37, 38). Combining immunophenotyping with transcriptomic, epigenetic, spatial, and functional analyses can narrow these alternatives, although no single cross-sectional assay establishes cellular ancestry.

The apparent disagreement between stable tissue-associated signatures and inducible inflammatory phenotypes is therefore partly a matter of timescale and experimental readout, rather than proof of mutually exclusive models. Cross-sectional human profiles identify states present at sampling; cytokine-culture experiments establish response potential; and mouse lineage tracing can test cellular ancestry under the conditions examined. Claims of durable conversion in human allergic tissue require longitudinal or perturbational evidence, not provided by marker shifts alone (3, 15, 45).

Cytokine-driven conversion in culture demonstrates potential, not its frequency or reversibility in patients. Intermediate states in inflamed human samples may reflect activation, selective recruitment, or marker modulation rather than conversion of an established resident cell. Mouse lineage tracing can more directly resolve ancestry, but species and tissue differences limit extrapolation. Claims of clinically relevant plasticity therefore require convergent longitudinal and functional evidence, rather than treating culture, lineage tracing, and single-cell profiles as interchangeable (3, 35, 45).

Developmental programming establishes lineage competence, tissue-derived signals refine mature identity, and functional plasticity permits adaptation to changing local conditions. These processes generate specialized ILC populations capable of integrating immune defense with the physiological requirements of individual organs. These principles are particularly relevant to ILC2s in allergic barrier tissues, where persistent epithelial and stromal signals can redirect protective responses toward chronic type 2 inflammation. The following section examines how tissue-adapted ILCs maintain epithelial integrity, coordinate tissue repair, and preserve homeostasis across barrier tissues.

Figure 1 summarizes the conventional developmental framework, subset-associated effector programs, and illustrative tissue distributions. Tissue adaptation and plasticity discussed above explain why these subset labels do not exhaust mature ILC diversity.

**Figure 1:**
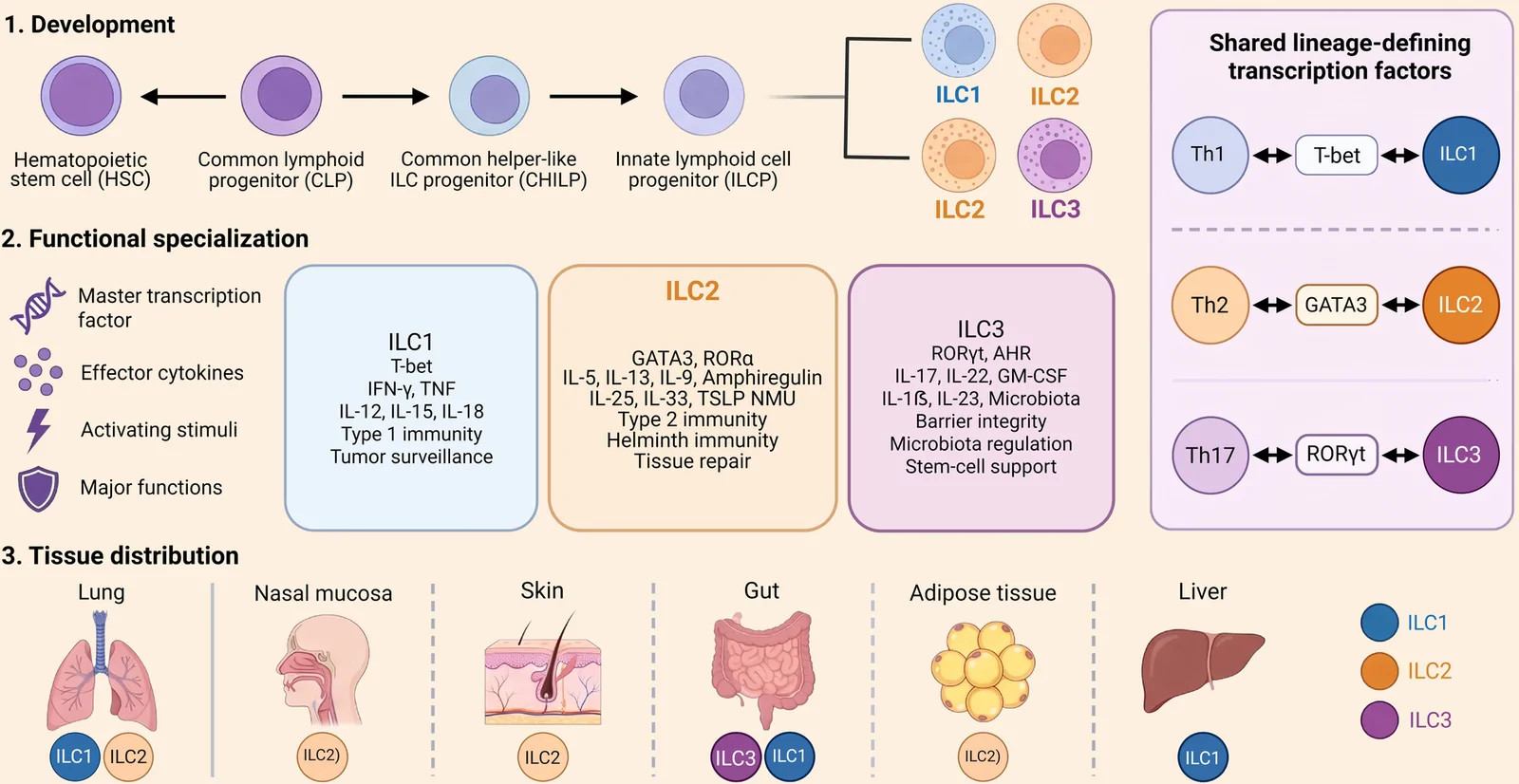
Development, tissue adaptation, and functional specialization of innate lymphoid cells. Innate lymphoid cells (ILCs) develop from hematopoietic stem cells (HSCs) through a simplified framework of common lymphoid progenitors (CLPs), common helper innate lymphoid progenitors (CHILPs), and innate lymphoid cell progenitors (ILCPs), which give rise to the three major ILC subsets: ILC1s, ILC2s, and ILC3s. Each subset is defined by distinct lineage-specifying transcription factors, activating signals, cytokine profiles, and specialized functions. ILC1s express T-bet and respond to IL-12, IL-15, and IL-18 by producing IFN-*γ* and TNF, thereby promoting type 1 immunity and tumor surveillance. ILC2s depend on GATA3 and ROR*α* and are activated by epithelial-derived alarmins (IL-25, IL-33, and TSLP) and neuromedin U (NMU), leading to the production of IL-5, IL-9, IL-13, and amphiregulin (AREG), which support type 2 immunity, anti-helminth responses, tissue repair, and metabolic homeostasis. ILC3s express ROR*γ*t and AHR and respond to IL-1β, IL-23, and microbial-derived signals by producing IL-17, IL-22, and GM-CSF, thereby maintaining epithelial barrier integrity, regulating host–microbiota interactions, and preserving tissue homeostasis. The lower panel illustrates the preferential tissue distribution of each ILC subset across major barrier and metabolic organs. The inset highlights the shared lineage-defining transcription factors between ILC subsets and their corresponding T helper cell lineages. The developmental arrows summarize conventional murine progenitor relationships rather than an obligatory sequence for every ILC, and do not depict the stem-cell-independent embryonic waves discussed in Section 2.1. Tissue distributions are illustrative rather than exclusive, and activating signals and effector functions vary with species and context (21, 22, 25, 26).

## 3 Innate lymphoid cells as coordinators of tissue homeostasis

Barrier tissues are continuously exposed to microorganisms, allergens, dietary antigens, environmental pollutants, and mechanical injury while maintaining essential physiological functions. Preserving tissue integrity under these conditions requires immune responses that rapidly control harmful stimuli while limiting collateral damage and restoring normal tissue architecture. Positioned within specialized tissue niches, innate lymphoid cells (ILCs) are well suited to coordinate these processes. Their localization adjacent to epithelial surfaces, blood vessels, peripheral nerves, fibroblasts, and stem-cell compartments enables them to detect changes in the local environment and communicate rapidly with structural and immune cells. Their functions therefore extend beyond early cytokine production to include maintenance of epithelial integrity, organization of multicellular tissue networks, integration of physiological signals, and restoration of tissue function after injury (1, 2, 51).

Unlike circulating leukocytes that enter tissues predominantly after injury, resident ILCs continuously receive signals from epithelial and stromal cells, neurons, metabolites, and the microbiota. This ongoing communication allows local immune activity to be adjusted to changes in tissue physiology before damage becomes extensive. However, the biological consequences of ILC activation depend strongly on context. Transient production of IL-13, IL-22, amphiregulin, and related mediators can support epithelial defense and regeneration, whereas persistent production promotes chronic inflammation, fibroblast activation, extracellular matrix deposition, and tissue remodeling. Tissue homeostasis therefore depends not only on initiating protective responses but also on their appropriate magnitude and timely termination (4, 52).

The following sections examine four interconnected mechanisms through which tissue-resident ILCs preserve homeostasis: maintenance of epithelial integrity, coordination of multicellular tissue niches, integration of neuroimmune and metabolic signals, and restoration of tissue function after injury. These functions are not separate activities but components of a coordinated tissue response that couples immune defense to repair and resolution.

### 3.1 Maintenance of epithelial barrier integrity, tissue repair, and regeneration

The epithelial barrier constitutes the first line of defense against environmental challenges and therefore requires continuous surveillance and renewal. In the respiratory tract, intestine, and skin, epithelial cells are exposed continuously to microorganisms, dietary antigens, allergens, pollutants, and mechanical stress. Preservation of barrier function depends on rapid communication among epithelial cells, stromal populations, resident immune cells, and the microbiota, allowing local protective and reparative responses to begin before tissue damage becomes extensive (1, 2, 51).

Epithelial cells actively regulate these responses through the release of alarmins, including IL-33, IL-25, and thymic stromal lymphopoietin (TSLP), together with lipid mediators, metabolites, and growth factors. At steady state, low-level tissue-derived signals contribute to ILC survival, positioning, and immune surveillance. Following injury, increased production or release of these mediators rapidly activates resident ILC populations, particularly ILC2s in response to epithelial alarmins, and initiates protective programs before adaptive immune responses are fully established. Epithelial cells therefore function not only as physical barriers but also as sensors that communicate tissue status to local immune networks (44, 53, 54).

Among the ILC subsets, ILC3s are central regulators of intestinal epithelial defense. Sanos and colleagues identified IL-22-producing NKp46⁺ innate lymphocytes in the intestinal mucosa (30). In murine bacterial infection, CD4⁺ lymphoid tissue inducer cells provided IL-22-dependent protection and promoted antimicrobial responses (55). IL-22 can also act directly on intestinal stem cells to support epithelial regeneration after injury (52). These findings connect innate immune sensing with epithelial protection, while the wider IL-22 literature shows that outcomes depend on tissue, inflammatory context, and duration of signaling (56).

Complementary reparative functions have been described for ILC2s in the respiratory tract. Monticelli and colleagues showed that lung-resident ILC2s accelerate recovery from influenza-induced epithelial injury through amphiregulin production independently of classical type 2 cytokines (4). Through epidermal growth factor receptor signaling, amphiregulin supports epithelial and stromal-cell survival and proliferation, facilitating restoration of tissue architecture. The subsequent identification of amphiregulin-producing regulatory T cells demonstrated that tissue repair is coordinated by several resident immune populations rather than controlled by a single cell type (57, 58). In helminth-induced lung injury, autocrine IL-9 supports ILC2 survival and damage control during recovery, demonstrating another context in which sustained ILC2 function can be protective (59). These infection models establish repair potential but do not imply that all ILC2 activation in allergic disease is reparative.

The outcome of these pathways is determined by their magnitude, timing, and duration. During acute injury, transient production of IL-13, IL-22, amphiregulin, and other repair mediators supports epithelial differentiation, mucus production, antimicrobial defense, and regeneration. As tissue integrity is restored, activating signals decline and reparative programs are disengaged. Persistent epithelial stress, by contrast, maintains ILC activation and converts regenerative signals into drivers of goblet-cell metaplasia, fibroblast activation, extracellular matrix deposition, and progressive remodeling (13, 60).

Maintenance of epithelial integrity therefore reflects a dynamic partnership between structural and resident immune cells. ILCs rapidly couple epithelial sensing to local defense and regeneration, but their contribution remains beneficial only when repair programs resolve after restoration of the barrier. Failure to terminate these responses establishes an early mechanistic link between physiological regeneration and chronic inflammatory remodeling.

### 3.2 ILCs coordinate multicellular tissue niches

Effective tissue homeostasis depends on continuous communication among structural, immune, vascular, and neuronal cells organized within specialized anatomical niches. ILCs reside at strategic positions where they interact with epithelial cells, fibroblasts, endothelial cells, resident macrophages, dendritic cells, regulatory T cells, and stem-cell compartments. Their positioning determines which combinations of cytokines, growth factors, metabolites, extracellular-matrix signals, and neuronal mediators they receive and therefore contributes directly to their survival and functional state (40, 44).

Structural cells form the foundation of these niches. Epithelial and stromal populations produce IL-7, IL-33, IL-25, TSLP, stem-cell factor, chemokines, growth factors, and extracellular-matrix components that regulate ILC maintenance, localization, and activation. ILC-derived IL-13, IL-22, amphiregulin, granulocyte–macrophage colony-stimulating factor (GM-CSF), and lymphotoxin in turn influence epithelial differentiation, fibroblast activity, extracellular-matrix turnover, and local myeloid-cell function. These reciprocal interactions allow tissues to adjust immune activity and repair in response to physiological stress (1, 40, 44).

Fibroblasts are increasingly recognized as active organizers of ILC niches rather than passive producers of extracellular matrix. In the intestine, Cheng and colleagues identified anatomically and functionally distinct fibroblastic reticular-cell populations that support different ILC compartments through specialized combinations of survival and positioning signals. Disruption of these stromal niches altered ILC maintenance and function, demonstrating that fibroblast identity and anatomical location directly regulate innate lymphoid homeostasis (40).

The relationship is reciprocal. ILC-derived cytokines can alter fibroblast activation, differentiation, and production of extracellular matrix, whereas fibroblast-derived mediators can stabilize or redirect ILC phenotype. In experimental arthritis and human synovial analyses, Rauber and colleagues identified a CD200⁺DKK3⁺ fibroblast population that interacted with CD200R1-expressing ILC2s and supported a pro-resolving tissue environment. This study provided direct evidence that fibroblast–ILC communication can actively promote resolution rather than merely support inflammation (61). In experimental silicosis, mechanically activated fibroblasts can alter ILC phenotype through Notch3- and IL-18-dependent signaling, illustrating how changes in the stromal environment may redirect ILC function during silica-associated chronic lung injury (62).

Communication with myeloid cells further refines these responses. Macrophages and dendritic cells sense microbial invasion and tissue injury and release IL-1β, IL-23, IL-12, TL1A, and other mediators that activate or redirect different ILC subsets according to the local challenge. Activated ILCs, in turn, influence myeloid-cell recruitment, antimicrobial activity, macrophage differentiation, and tissue repair. These interactions can amplify protective immunity but also provide inhibitory and resolving signals that limit unnecessary tissue damage (1, 49, 63).

ILCs also shape adaptive immunity through subset-specific interactions with lymphocytes. Amphiregulin-producing regulatory T cells (Tregs) can support tissue protection, although this does not establish that ILC2-derived amphiregulin is the signal driving Treg accumulation (57, 58). In experimental arthritis, IL-9-producing ILC2s promote resolution through interactions with Tregs, providing evidence for an ILC2–regulatory-cell circuit outside allergic disease (64). In the intestine, major histocompatibility complex (MHC) class II expression by subsets of ILC3s limits commensal-reactive CD4⁺ T-cell responses, contributing to host–microbiota mutualism (65, 66). By contrast, MHC class II-dependent communication between ILC2s and CD4⁺ T cells promotes type 2 immunity in murine helminth infection, and ILC2-derived IL-4 supports Th2 differentiation in a separate helminth model (67, 68). Thus, ILC–T-cell interactions can restrain or enhance adaptive responses according to subset and context; the infection results should not be treated as direct demonstrations of the same mechanisms in human allergy.

The vascular compartment supplies oxygen and nutrients and regulates leukocyte entry. Its relationship to ILC niches requires a distinction between anatomical proximity and demonstrated signaling. Murine lung studies identify perivascular adventitial stromal niches associated with ILC2s, while circulating human progenitors provide a potential source of tissue ILCs (35, 44). These findings support a role for vascular position in access to tissue signals, but do not establish that ILCs directly control angiogenesis or perfusion in human allergic barriers.

Recent single-cell and spatial studies have reinforced the importance of anatomical context. Cross-tissue analysis of human ILCs identified conserved lineage-associated features together with strong tissue-specific transcriptional imprinting in blood, lymphoid organs, mucosal tissues, inflammatory lesions, and tumors (38, 39). Analysis of human bone marrow, spleen, and lymph nodes similarly revealed marked differences in ILC composition and maturation state among anatomical sites (39).

Spatial mapping has begun to localize these populations directly within intact human tissue. Van Acker and colleagues developed a multiplex histological approach that distinguished ILC subsets in human lymphoid tissues and demonstrated non-random positioning and preferential proximity to functionally corresponding T-cell populations (39). Such studies show that ILC identity cannot be understood fully from dissociated-cell transcriptional profiles alone because spatial position determines the cellular partners and regulatory signals available to each population.

ILCs should therefore be viewed as participants in multicellular tissue niches rather than isolated cytokine-producing cells. Fibroblasts, epithelial cells, endothelial cells, neurons, and resident immune populations jointly establish these niches, while ILCs integrate and redistribute signals among their components. This organization provides the structural basis through which physiological, neuronal, and metabolic cues regulate local immunity.

The niche studies also illustrate an important distinction between disease settings: fibroblast–ILC communication may accompany resolution in arthritis but support inflammatory plasticity in silicosis. Both provide mechanistic examples of stromal instruction, yet neither demonstrates an equivalent circuit in human allergic barriers. Future allergy studies should therefore pair spatial localization with pathway perturbation and repair outcomes (61, 62).

### 3.3 Integration of neuroimmune and metabolic signals

Barrier tissues function within dynamic physiological environments shaped by neuronal activity, nutrient availability, microbial metabolism, circadian rhythms, and oxygen tension. Maintaining homeostasis therefore requires immune cells to interpret non-immunological cues and adjust their responses without compromising barrier function. Owing to their tissue residency and broad repertoire of environmental receptors, ILCs integrate these physiological signals with immune and regenerative programs (2).

The peripheral nervous system actively regulates tissue immunity. Barrier organs are densely innervated by sensory, sympathetic, parasympathetic, and enteric neurons that monitor mechanical stress, microbial products, nutrients, and tissue injury. Resident ILCs express receptors for multiple neuropeptides and neurotransmitters, allowing neuronal circuits to alter their proliferation, cytokine production, migration, and metabolic activity (14, 41, 69).

Klose and colleagues and Wallrapp and colleagues demonstrated that neuromedin U, released by enteric and sensory neurons, activates ILC2s through neuromedin U receptor 1. Neuromedin U (NMU) rapidly induces ILC2 proliferation and IL-5 and IL-13 production, enhancing immunity against helminths and coordinating local changes in epithelial physiology (14, 70). These studies established that neuronal signals can directly initiate innate immune responses rather than acting only through secondary inflammatory pathways.

Excitatory neuronal signaling is balanced by inhibitory circuits. Catecholamines released by sympathetic neurons suppress ILC2 proliferation and cytokine production through β_2_-adrenergic receptors, providing a physiological restraint on type 2 immunity (41). Vasoactive intestinal peptide links feeding and circadian behavior to intestinal ILC3 function and IL-22 production, whereas calcitonin gene-related peptide modulates ILC2 activity in a tissue- and context-dependent manner (69, 71–73). Neuronal regulation therefore contributes both activating and inhibitory signals that calibrate ILC responses according to tissue activity.

Metabolic signals provide another level of regulation. Resident ILCs adapt to changes in nutrient availability, microbial metabolites, oxygen tension, and systemic energy balance. In adipose tissue, ILC2-derived IL-5 and IL-13 support eosinophils and alternatively activated macrophages, contributing to an anti-inflammatory tissue environment (74). ILC2-associated pathways have also been linked to beige adipocyte differentiation, thermogenesis, and energy expenditure, connecting tissue-resident immunity with systemic metabolism (75, 76). ILC2-derived IL-5 also contributes to eosinophil maintenance under steady-state conditions in mice, separating a homeostatic function from allergen-induced eosinophilia (77). A brain–adipose neuro-mesenchymal circuit provides an additional example of neural and stromal control of ILC2s, although its relevance to allergic barriers requires direct testing (78).

Dietary nutrients and microbiota-derived metabolites further shape ILC function. Vitamin A deficiency reduces IL-22-producing ILC3s while favoring ILC2-associated responses, altering intestinal defense and epithelial integrity (79). Activation of the aryl hydrocarbon receptor by dietary and microbial tryptophan metabolites supports ILC3 maintenance and IL-22 production (42, 80). Fatty-acid utilization also supports ILC2-mediated barrier protection during malnutrition and helminth infection in mice, illustrating a metabolic requirement that should be distinguished from an established treatment target in allergy (43).

Oxygen availability is particularly important in the intestinal mucosa, where steep oxygen gradients exist under physiological conditions and change during inflammation. Hypoxia-inducible factor 1*α* (HIF-1α)-dependent programs influence ILC3 cytokine production and metabolic adaptation, linking oxygen sensing with epithelial antimicrobial defense and regeneration (81). Metabolic regulation of ILCs therefore reflects not merely the energetic requirements of activated cells but a mechanism through which tissue physiology shapes immune function.

Neuronal, metabolic, microbial, and circadian signals continuously calibrate ILC activity according to the requirements of individual tissues. These pathways influence not only whether an ILC response begins, but also its location, intensity, duration, and interaction with structural cells. Successful homeostasis consequently depends on the coordinated engagement and withdrawal of these signals as tissue conditions change.

### 3.4 Resolution and restoration of tissue homeostasis

Effective tissue homeostasis depends on timely restraint of inflammation as injury resolves. Resolution is an active process involving regulatory pathways and clearance of inflammatory cells rather than passive disappearance of inflammation (82). Reconstruction of tissue architecture additionally depends on coordinated repair by immune and structural cells [Wynn and Vannella, (63)]. Restoration of local metabolic and microbial conditions is part of the broader homeostatic framework considered here; it should not be inferred from a resolution citation alone.

Recovery requires changes in the signals that maintain inflammatory activity. Regulatory macrophage states can limit damaging inflammation and support tissue reconstruction, but macrophages can also promote fibrosis when injury persists (63). IL-33 has context-dependent effects on inflammatory and reparative populations, and its trajectory during recovery cannot be assumed to be identical across tissues (53). Accordingly, withdrawal of epithelial stimulation is a proposed component of the failed-homeostasis model, rather than a uniform sequence demonstrated for IL-33, IL-25, and TSLP in every allergic disease.

Tissue-resident Tregs contribute to protection through amphiregulin, while an IL-33–ILC2–amphiregulin pathway supports intestinal tissue protection through epidermal growth factor receptor (EGFR) signaling (57, 83). These studies establish complementary repair mechanisms in different experimental settings. Evidence that IL-9-producing ILC2s support Treg-associated resolution comes from arthritis models, and its operation in allergic barriers remains to be established (64).

Fibroblasts also participate actively in resolution. In arthritis, the identification of CD200⁺DKK3⁺ fibroblasts that stabilize ILC2 identity and support resolution demonstrates that stromal cells can acquire pro-resolving states rather than serving only as sources of inflammatory mediators or extracellular matrix (61). This observation broadens the concept of resolution from suppression of leukocyte activity to coordinated reprogramming of both immune and structural compartments.

Physiological inhibitory pathways provide additional restraint. Β_2_-adrenergic signaling and calcitonin gene-related peptide (CGRP) can restrain ILC2 responses in defined experimental settings (41, 71, 72). Restoration of epithelial integrity, vascular function, oxygen balance, nutrient availability, and microbial composition progressively removes the environmental signals that initiated repair. Resolution thus requires the return of the entire tissue niche to a stable state.

Persistent epithelial injury and altered regulatory circuits can sustain inflammatory signaling and interfere with return to homeostasis. IL-5 supports eosinophil responses, whereas IL-13 can contribute to mucus metaplasia and profibrotic signaling. Amphiregulin and IL-22 have protective effects in some injury models, but their activities depend on tissue and inflammatory context; IL-17 should not be grouped with them as a uniformly reparative mediator (4, 13, 56, 77). Fibrosis literature supports a role for persistent inflammatory and growth-factor signaling in matrix accumulation, without establishing that every listed mediator or ILC subset drives fibrosis in each allergic organ (60).

Failure of resolution may contribute to the transition between physiological tissue repair and chronic inflammatory disease. ILCs participate in this transition not because their homeostatic functions are inherently pathogenic, but because the epithelial, stromal, neuronal, metabolic, and immune signals that normally constrain those functions remain persistently altered. Repeated injury thereby converts a transient multicellular repair response into a stable pathological tissue niche.

Throughout this section, ILCs have emerged as coordinators of epithelial maintenance, multicellular communication, physiological integration, repair, and resolution. Disruption of these interconnected processes prevents restoration of barrier homeostasis and sustains the ILC2-associated pathways that contribute to chronic allergic inflammation. The following section examines how persistent epithelial injury and failed resolution transform these physiological functions into drivers of asthma, chronic rhinosinusitis with nasal polyps, eosinophilic gastrointestinal disease, atopic dermatitis, fibrosis, and progressive organ dysfunction.

Figure 2 illustrates the multicellular circuits through which ILC subsets contribute to barrier maintenance, repair, and resolution.

**Figure 2:**
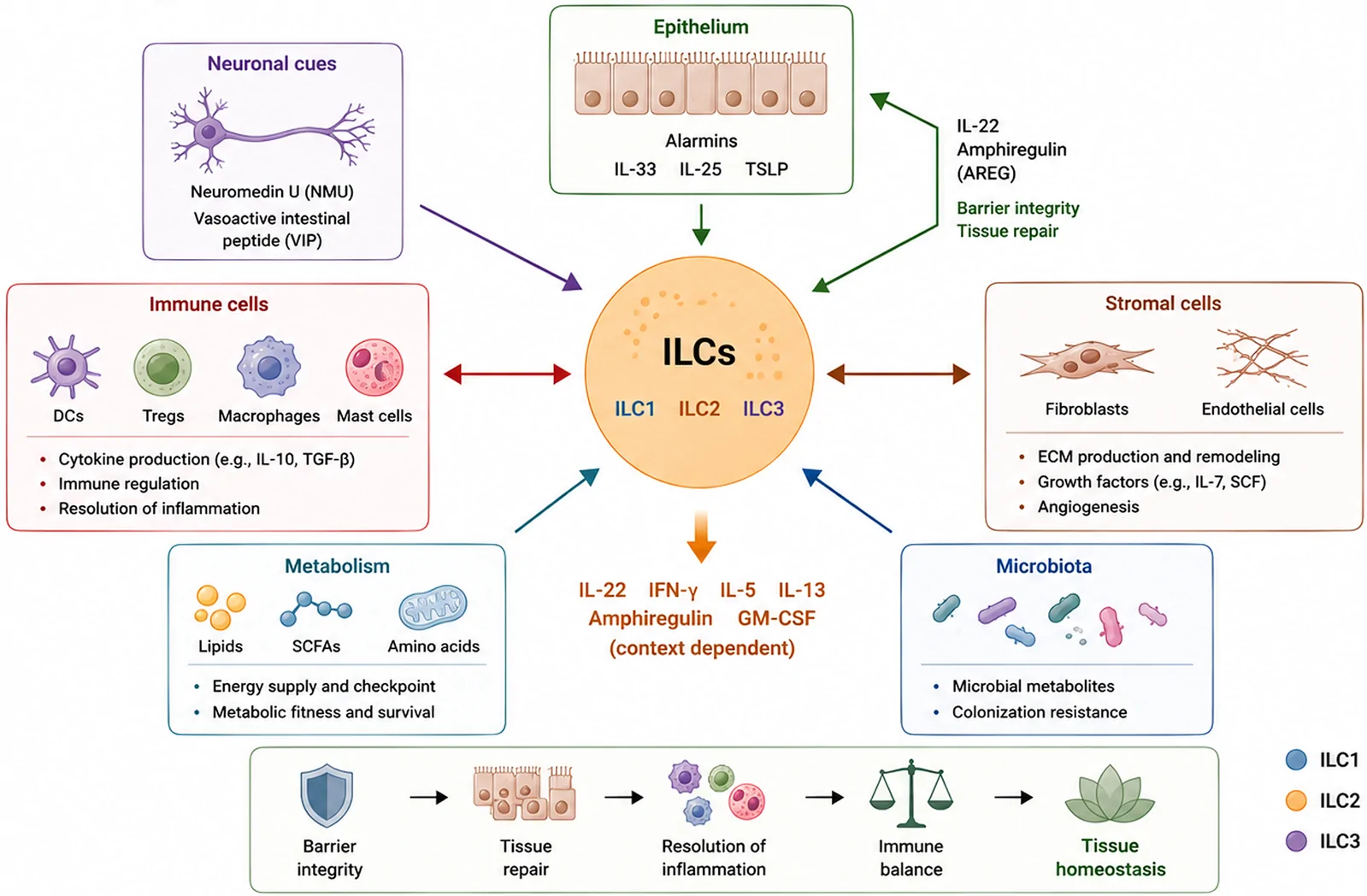
ILCs as guardians of tissue homeostasis. ILCs integrate signals from epithelial, stromal, immune, neuronal, microbial, and metabolic compartments. Epithelial IL-25, IL-33, and TSLP particularly activate ILC2s; ILC3-derived IL-22 and ILC2-associated amphiregulin can support epithelial protection and repair in appropriate contexts. Neuronal mediators including neuromedin U and vasoactive intestinal peptide regulate particular ILC subsets. Interactions with dendritic cells, regulatory T cells, macrophages, mast cells, fibroblasts, and endothelial cells contribute to immune regulation and tissue repair. Metabolites and nutrient cues further shape ILC survival and function. These processes depend on subset, tissue, and duration of stimulation.

## 4 Allergic diseases: when homeostatic programs fail

### 4.1 From physiological homeostasis to chronic inflammation

The tissue-protective functions of innate lymphoid cells (ILCs) are tightly controlled in both magnitude and duration to preserve barrier integrity while minimizing collateral tissue damage. As discussed in the previous section, tissue-resident ILCs continuously communicate with epithelial, stromal, neuronal, vascular, and immune cells to coordinate immune surveillance, tissue repair, and restoration of organ function. Once the barrier is repaired, these responses subside and the tissue returns to homeostasis (1–3, 84).

Chronic allergic diseases arise when these repair programs are activated repeatedly but fail to resolve. Ongoing exposure to allergens, respiratory viruses, environmental pollutants, microbial dysbiosis, or mechanical injury maintains epithelial stress and sustained production of the alarmins IL-33, IL-25, and thymic stromal lymphopoietin (TSLP). Rather than acquiring new functions, ILC2s and other tissue-resident immune cells may prolong programs normally associated with barrier repair and tissue recovery. When these responses persist, however, prolonged production of type 2 cytokines and repair-associated mediators drives eosinophilic inflammation, mucus hypersecretion, fibroblast activation, extracellular matrix deposition, and progressive remodeling of the affected tissue (4, 13, 52, 58, 60). Recent studies further demonstrate that the magnitude and persistence of these responses are shaped by tissue-specific stromal niches, neuronal inputs, and local metabolic cues, emphasizing that ILC function is determined by the surrounding microenvironment rather than by cytokine stimulation alone (85, 86).

Repeated tissue injury also reshapes the local cellular environment. Activated epithelial cells, fibroblasts, endothelial cells, neurons, and infiltrating immune cells establish reciprocal interactions that sustain inflammatory and repair signals while preventing restoration of normal tissue architecture. In this setting, inflammation is no longer simply a protective response to injury but a consequence of repair processes that remain chronically engaged. Advances in single-cell and spatial profiling have further shown that chronic allergic inflammation is maintained within specialized multicellular niches, where structural cells and resident immune populations cooperate to reinforce local type 2 responses and tissue remodeling. Viewed from this perspective, failure to restore tissue homeostasis may reinforce immune dysregulation in allergic disease (12, 44, 87, 88).

The concept of failed restoration of tissue homeostasis does not imply that allergic diseases follow a single uniform pathogenic mechanism. Asthma, chronic rhinosinusitis with nasal polyps, eosinophilic esophagitis, and atopic dermatitis differ in initiating stimuli, epithelial architecture, microbial and environmental exposures, contributions from adaptive immunity, clinical phenotype, and responses to therapy. Rather, failed homeostatic restoration offers a shared conceptual framework in which recurrent or persistent tissue injury can sustain inflammatory and repair-associated circuits. The molecular components and relative contribution of ILC2s are disease- and tissue-dependent. The following sections therefore examine both shared epithelial alarmin–ILC2 pathways and the organ-specific networks that shape each disease (2, 89, 90).

Figure 3 presents the proposed failed-homeostasis framework as a conceptual synthesis, not a claim that all allergic diseases share identical initiating events or an equally established ILC2 contribution.

**Figure 3:**
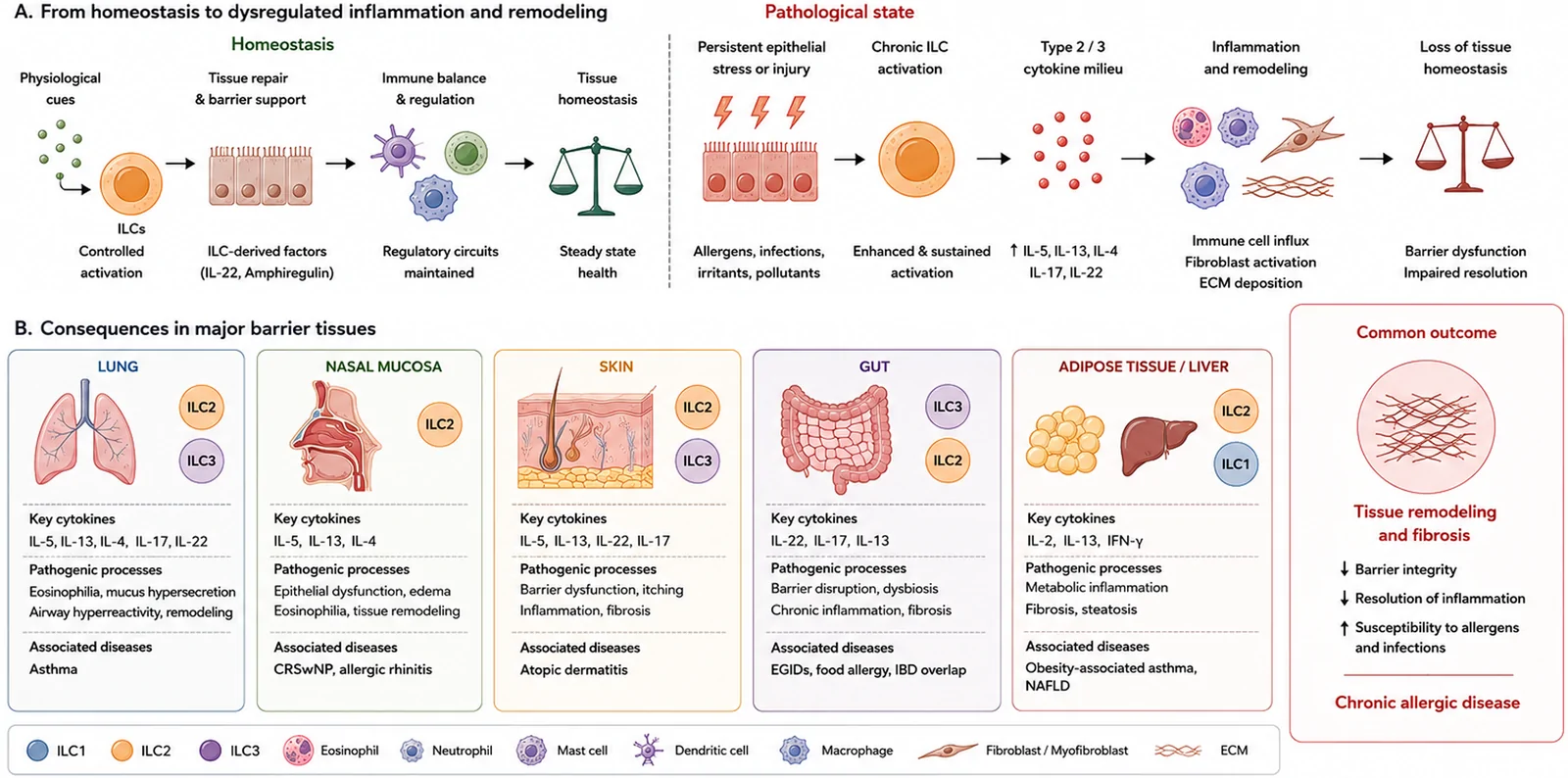
Loss of tissue homeostasis drives allergic disease. **(A)** Under homeostatic conditions, controlled responses by tissue-resident ILC subsets support epithelial barrier maintenance, repair, and immune regulation. Persistent injury may sustain ILC2 activation and IL-5/IL-13-associated type 2 inflammation; ILC3s and plastic states can contribute IL-17- or IL-22-associated responses in context-dependent settings. Interactions with infiltrating leukocytes and structural cells may promote extracellular matrix deposition and remodeling. **(B)** Asthma, chronic rhinosinusitis with nasal polyps, eosinophilic esophagitis, and atopic dermatitis share some epithelial alarmin and type 2 pathways but differ in initiating stimuli, tissue architecture, adaptive immune contributions, and clinical manifestations. Failed homeostatic restoration is presented as a conceptual framework, not a uniform mechanism or proof of ILC2 causality in every disease. Panel B also includes adipose and liver examples as broader metabolic context. Listed cytokines describe the local inflammatory milieu and are not all assigned to ILC2s; IL-4 can derive from several populations. Remodeling and fibrosis are possible context-dependent outcomes, not inevitable or equivalent features of every disease shown.

### 4.2 Asthma

Asthma encompasses heterogeneous airway diseases with variable airflow obstruction, hyperresponsiveness, and, in some patients, persistent remodeling. In type 2 disease, epithelial IL-33, IL-25, and TSLP can activate ILC2s and connect environmental injury to eosinophilic inflammation (85, 91, 92). Amphiregulin-mediated repair has been demonstrated after influenza infection, whereas IL-5 and IL-13 promote eosinophilic and mucus-associated responses in allergic models; these outputs should not all be described as equivalent repair programs. Repeated injury may sustain these circuits, but ILC2 activity does not explain all non-type 2 or mixed asthma phenotypes.

The discovery of ILC2s in 2010 fundamentally changed the understanding of type 2 immunity. Three independent groups almost simultaneously identified previously unrecognized innate lymphoid populations that responded to epithelial-derived cytokines and produced large quantities of IL-5 and IL-13 despite lacking antigen-specific receptors. Neill and colleagues described natural helper cells responsive to IL-33, Moro and colleagues identified fat-associated lymphoid cluster cells that promoted type 2 immunity, while Price and Saenz independently demonstrated IL-25-responsive innate lymphoid populations capable of driving eosinophilic inflammation in the absence of adaptive immunity (6–8, 93). These landmark discoveries established that innate lymphoid cells constitute a rapid tissue-resident source of type 2 cytokines and provided a mechanistic explanation for allergic inflammation occurring independently of conventional Th2-cell activation.

The relevance of these findings to asthma rapidly became apparent through experimental models of allergic airway disease. Bartemes et al. first demonstrated that IL-33 administration induced robust expansion of pulmonary ILC2s, leading to eosinophilic airway inflammation and airway hyperresponsiveness through rapid production of IL-5 and IL-13 (94) [Bartemes et al., (94)]. Shortly thereafter, Klein Wolterink et al. showed that pulmonary ILC2s accumulated following influenza infection and allergen exposure, functioning as an important early source of type 2 cytokines within the lung. Their work further demonstrated that depletion of ILC2s markedly reduced eosinophilic inflammation and airway hyperreactivity, establishing these cells as critical initiators of allergic airway responses rather than passive amplifiers of adaptive immunity (95).

Subsequent studies using clinically relevant allergen models further expanded this concept. Halim et al. demonstrated that intranasal papain rapidly activated resident pulmonary ILC2s through epithelial-derived IL-33, resulting in eosinophilic inflammation even in Rag-deficient mice lacking functional T and B lymphocytes. In addition to initiating innate type 2 responses, activated ILC2s promoted dendritic-cell migration and enhanced Th2-cell priming, thereby linking early epithelial injury with subsequent adaptive immunity (91). Similar observations were reported in models using Alternaria alternata, house dust mite, fungal proteases, and recombinant epithelial alarmins, supporting a role for ILC2s in several experimental responses to epithelial injury while leaving the relative contribution of other innate and adaptive pathways dependent on the exposure (91, 94).

Murine studies also revealed that ILC2s integrate multiple epithelial-derived signals rather than responding exclusively to a single cytokine. IL-33 is a potent activator of resident natural ILC2s, whereas IL-25 preferentially expands inflammatory ILC2 populations capable of migrating between tissues and differentiating in response to changing cytokine environments (96). TSLP further enhances ILC2 proliferation, survival, and cytokine production while simultaneously promoting resistance to regulatory mechanisms that normally limit tissue inflammation. Together, these epithelial cytokines create a coordinated alarmin network that allows ILC2s to respond rapidly to environmental injury while simultaneously providing functional redundancy that ensures continued tissue protection even when individual signaling pathways are disrupted.

As experimental models became more sophisticated, it became increasingly apparent that ILC2s contribute not only to the initiation of airway inflammation but also to its persistence. A particularly important advance came from Christianson and colleagues, who demonstrated that established allergic airway disease is maintained through interacting feedback circuits involving IL-33, ILC2s, and adaptive lymphocytes rather than continued activation of a single inflammatory pathway (97). Using a chronic allergen model, they showed that elimination of both T cells and ILC2s or blockade of IL-33 permitted airway inflammation and airway hyperresponsiveness to resolve, whereas interruption of individual downstream mediators produced only partial improvement. These findings shifted attention from the initiation of asthma toward the mechanisms responsible for failed resolution, identifying the IL-33–ILC2 axis as a central component of self-sustaining repair circuits that maintain chronic airway inflammation after repeated epithelial injury.

This concept was further strengthened by Verma et al., who demonstrated that experimental asthma persisted even in mice lacking the IL-33 receptor ST2 because alternative TSLP-driven IL-9⁺ and IL-13⁺ ILC2 populations emerged to sustain airway inflammation (92). Rather than functioning as isolated signaling pathways, IL-33 and TSLP therefore operate within an interconnected epithelial cytokine network capable of compensating for the loss of individual components. These findings provided an important mechanistic explanation for the limited efficacy sometimes observed with blockade of single epithelial alarmins and highlighted the remarkable functional plasticity of tissue-resident ILC2s during chronic allergic inflammation.

The mechanistic observations made in experimental models were subsequently confirmed in patients with asthma, where multiple independent studies established that ILC2s accumulate within the airways and contribute to disease severity. Increased numbers of activated ILC2s have been identified in bronchoalveolar lavage fluid, sputum, bronchial biopsies, and peripheral blood from patients with eosinophilic asthma, with the greatest expansion generally observed in severe disease. These studies confirmed that human ILC2s respond directly to epithelial-derived IL-33, IL-25, and TSLP and rapidly produce IL-5 and IL-13 independently of antigen recognition, supporting the concept that innate immune activation precedes and amplifies adaptive type 2 responses (94, 98).

As human studies expanded, it became apparent that the ILC2 compartment was considerably more heterogeneous than originally appreciated. Using additional phenotypic markers, Liu et al. identified both conventional CRTH2 + IL-7R*α*+ ILC2s and previously unrecognized CRTH2-IL-7R*α*- populations capable of producing type 2 cytokines in response to epithelial alarmins (37). These findings demonstrated that commonly used flow cytometric strategies substantially underestimate the complexity of human ILC2 populations and suggested that functionally important subsets may be overlooked when analyses rely exclusively on CRTH2 expression. Recognition of this heterogeneity has important implications for interpreting human studies and may partly explain the variability in ILC2 frequencies reported among different asthma cohorts.

One of the most clinically significant advances in the field has been the recognition that airway ILC2s contribute to corticosteroid resistance, a defining feature of severe asthma. Early work by Kabata and colleagues demonstrated that TSLP protected human ILC2s from glucocorticoid-mediated suppression through activation of STAT5 signaling, providing the first mechanistic link between epithelial alarmins and steroid-resistant innate immunity (99). Building on these observations, Liu et al. examined airway ILC2s obtained from patients with severe asthma and demonstrated that dexamethasone only partially suppressed cytokine production in the presence of TSLP. Mechanistically, TSLP activated MEK and STAT5 signaling pathways that restored cytokine production despite steroid treatment, whereas pharmacological inhibition of these pathways largely recovered glucocorticoid sensitivity (100). These findings established that epithelial-derived cytokines actively reprogram ILC2 responsiveness rather than simply overwhelming anti-inflammatory therapy, providing a mechanistic explanation for persistent eosinophilic inflammation despite high-dose corticosteroid treatment.

Experimental studies have since shown that steroid resistance reflects coordinated survival and metabolic programs rather than isolated cytokine signaling. Recent murine studies demonstrated that combined IL-33, TSLP, and IL-7 signaling activates JAK–STAT5-dependent expression of the anti-apoptotic molecule Bcl-xL together with PI3K–Akt–mTOR pathways, promoting survival of activated ILC2s and reducing glucocorticoid-induced apoptosis (101). These observations indicate that prolonged exposure to epithelial alarmins not only enhances cytokine production but also establishes cellular programs that maintain activated ILC2 populations during chronic airway inflammation.

Verma et al. identified nuclear factor *κ*B1 (NF*κ*B1) as a regulator that restrains memory-like ILC2 responses while supporting immediate effector function in experimental memory-driven asthma, distinguishing the molecular regulation of recall responses from acute cytokine production (102). Extending these observations, Sirohi et al. identified the chromobox protein CBX7 as a methylation-dependent regulator of cytokine transcription and cytosolic signaling in lymphoid cells. Genetic deletion or pharmacological inhibition of CBX7 markedly reduced cytokine production by murine lymphoid cells and lymphoid cells isolated from patients with asthma while suppressing allergic airway inflammation in multiple experimental models (103). Together, these studies suggest that persistent ILC2 activation is sustained not only by continued epithelial stimulation but also by stable transcriptional and cytosolic signaling that preserve cytokine competence after the initiating stimulus has diminished.

These mechanistic studies should not be generalized across all asthma endotypes. Mouse allergen challenges can isolate a defined pathway, whereas human asthma involves variable treatment exposure, chronicity, and overlapping type 2 and non-type 2 processes. A change in ILC2 frequency or cytokine output in a patient sample establishes association; the relative contribution of ILC2s vs. Th2 cells, eosinophils, and myeloid populations requires cell-resolved functional evidence (100, 104).

Although asthma has traditionally been viewed as a type 2 eosinophilic disease, recent work suggests that ILC2s also participate in broader inflammatory networks. Sripada et al. demonstrated that allergen exposure can activate macrophage stimulator of interferon genes (STING) signaling, leading to tumor necrosis factor *α* (TNF-α) production that cooperates with ILC2-derived cytokines to promote neutrophilic airway inflammation (104). These findings extend the functional repertoire of ILC2s beyond classical eosinophilic asthma and illustrate how the surrounding inflammatory environment shapes ILC function and disease phenotype. Rather than existing as fixed effector populations, tissue-resident ILC2s appear capable of integrating epithelial, myeloid, and stromal signals to generate context-dependent immune responses.

Increasing evidence also indicates that ILC2s communicate extensively with structural and neuronal cells that regulate airway physiology. Neuromedin U released from cholinergic neurons potently activates pulmonary ILC2s, whereas calcitonin gene-related peptide, vasoactive intestinal peptide, and adrenergic signaling modulate their proliferation and cytokine production, demonstrating that neural circuits directly influence innate type 2 immunity (2, 70, 78). Likewise, reciprocal interactions with airway epithelial cells, fibroblasts, smooth muscle cells, mast cells, macrophages, and eosinophils establish multicellular tissue niches that coordinate both immune activation and tissue repair. During acute injury these interactions facilitate restoration of epithelial integrity; however, repeated epithelial damage prolongs these communication networks, promoting goblet-cell metaplasia, smooth muscle hypertrophy, extracellular matrix deposition, and progressive airway remodeling. Thus, ILC2s contribute to chronic asthma not because their repair functions are inherently pathogenic, but because physiological repair programs remain chronically engaged in response to unresolved epithelial injury.

These observations have therapeutic implications. Approved biologics targeting IL-5, IL-5Rα, IL-4Rα, and TSLP interrupt type 2 inflammatory pathways in selected asthma populations; tezepelumab acts upstream by neutralizing epithelial-derived TSLP. IL-33/ST2-directed approaches remain investigational. Because these pathways are shared by ILC2s, Th2 cells, and other immune or structural cells, clinical responses do not establish ILC2-specific efficacy. Established airway remodeling may persist despite improved inflammatory control, motivating further study of barrier restoration and stromal–immune communication (105–109).

### 4.3 Chronic rhinosinusitis with nasal polyps: persistent mucosal remodeling in the upper airways

Chronic rhinosinusitis with nasal polyps (CRSwNP) provides a human setting in which epithelial dysfunction and type 2 inflammation can coexist with local ILC2 accumulation. IL-33-responsive cells in nasal polyps can produce IL-13, and human ILC2s respond to IL-25 and IL-33 (54, 110). These observations support an epithelial–ILC2 connection, but they do not establish that every CRSwNP endotype results from failed ILC2-mediated repair.

Human studies report enrichment and activation of ILC2s in nasal polyps, with associations involving allergy and inflammatory features (54, 111, 112). These cross-sectional findings establish relevance to diseased tissue, but cannot determine whether ILC2 accumulation precedes polyp formation or follows a niche already altered by adaptive immunity and stromal responses. ILC2 abundance should therefore be interpreted alongside functional measurements rather than as an independent demonstration of causality or a validated predictor of postoperative recurrence.

ILC2-associated IL-5 and IL-13 provide plausible links to eosinophilic inflammation and epithelial dysfunction, but these cytokines also arise from other cells. Nasal polyps contain interacting immune and structural populations, so their edema and remodeling cannot be attributed to ILC2s from cytokine expression alone (13, 110, 112). Claims that a particular fibroblast state directly sustains ILC2 activation require matched localization and perturbation evidence in sinonasal tissue.

The failed-homeostasis framework suggests that persistence of epithelial and stromal abnormalities may help maintain disease after removal of a polyp. This remains a mechanistic interpretation, rather than proof of a spatially defined ILC2–fibroblast circuit. Human intervention trials establish that targeting shared type 2 pathways can improve severe CRSwNP; they do not identify which cell type is responsible for the clinical response or demonstrate complete restoration of tissue architecture (113–116).

Biologics targeting IL-4R*α*, IL-5, IgE, and TSLP have demonstrated clinical benefit in appropriately selected patients with severe CRSwNP; dupilumab, mepolizumab, omalizumab, and tezepelumab have US approvals for this indication. IL-33/ST2 blockade remains investigational. These agents target shared inflammatory pathways rather than ILC2s specifically, and responses vary with disease endotype and patient characteristics. Persistent or recurrent disease highlights the need to understand epithelial and stromal contributions to mucosal remodeling (113–116).

### 4.4 Eosinophilic gastrointestinal diseases: loss of mucosal homeostasis at the gastrointestinal barrier

The gastrointestinal tract presents a unique challenge to the immune system. Unlike the airways or skin, gastrointestinal mucosal surfaces are continuously exposed to dietary antigens, commensal microorganisms, and microbial metabolites while maintaining epithelial integrity and immune tolerance. This balance depends on continuous communication among epithelial cells, stromal cells, resident immune populations, and the intestinal microbiota. Tissue-resident innate lymphoid cells contribute to this process by supporting epithelial renewal, reinforcing barrier function, and promoting repair following mucosal injury. When these regulatory mechanisms are disrupted, physiological repair responses can evolve into persistent inflammation and tissue remodeling (52, 56, 83).

Eosinophilic gastrointestinal diseases (EGIDs), including eosinophilic esophagitis (EoE), are heterogeneous conditions and should not be treated as a single ILC2-driven process. In EoE, food-antigen responses, epithelial barrier abnormalities, and type 2 cytokine signaling contribute to disease. Human biopsies show ILC2 enrichment during active disease, supporting their participation while leaving their relative contribution to adaptive and structural-cell pathways unresolved (117). Evidence from intestinal ILC3 repair models provides biological context but does not directly establish the mechanism of esophageal remodeling.

Evidence linking ILC2s to EoE emerged soon after their role in allergic airway disease became apparent. Doherty and colleagues identified an expanded population of ILC2s in esophageal biopsies from patients with active EoE and showed that their abundance correlated with tissue eosinophilia and disease activity (117). These human data support an association between ILC2 accumulation and active disease, but they do not establish that ILC2s are the dominant source of type 2 cytokines in the esophagus or that their expansion is sufficient to drive fibrostenosis. Broader mechanistic work has instead placed epithelial barrier dysfunction, IL-13-responsive epithelial programs, eosinophils, mast cells, and stromal remodeling within an interconnected disease network (89). More recent single-cell profiling of active and remitting EoE resolved epithelial, stromal, myeloid, lymphoid, and rare ILC2 states within the same tissue and identified disease-associated shifts in cell composition and inferred cell-cell communication (118). Together, these studies support a multicellular model in which ILC2s participate in type 2 inflammation but should not be considered in isolation from adaptive and structural-cell programs.

IL-13-responsive epithelial programs, eosinophils, mast cells, and stromal responses contribute to the EoE disease network and can accompany basal-zone hyperplasia and fibrostenotic change (89). Single-cell profiles further resolve multiple immune and structural populations, but inferred communication does not establish that ILC2-derived cytokines are the principal cause of fibrosis (118). Inflammation and established luminal narrowing therefore require distinct assessments; reduction of eosinophils alone cannot be assumed to reverse all structural disease.

Current therapeutic approaches—including dietary elimination, proton pump inhibitors, swallowed topical corticosteroids, and biologic therapy targeting IL-4R*α*—effectively reduce eosinophilic inflammation in many patients. The clinical efficacy of dupilumab has provided direct evidence that interrupting type 2 cytokine signaling improves both histologic inflammation and clinical symptoms (119), supporting a role for IL-4/IL-13 signaling without establishing that the clinical benefit is mediated specifically through ILC2s. Clinical improvement does not by itself demonstrate complete reversal of established fibrosis; structural outcomes should be assessed separately (89). Future therapies will likely need to combine control of type 2 inflammation with strategies that restore epithelial barrier function, re-establish immune tolerance to dietary antigens, and promote resolution of tissue repair. Similar principles are evident in the skin, where repeated barrier disruption can sustain type 2 inflammation involving ILC2s and other populations and contributes to the chronic course of atopic dermatitis.

### 4.5 Atopic dermatitis: persistent barrier dysfunction and neuroimmune amplification

The skin forms the body's largest barrier against the external environment and is continuously challenged by allergens, microbes, environmental irritants, and mechanical injury. Maintaining epidermal integrity requires coordinated communication among keratinocytes, fibroblasts, resident immune cells, sensory neurons, vascular cells, and the skin microbiota. Tissue-resident ILC2s contribute to this process by responding rapidly to epithelial damage and promoting repair while limiting unnecessary inflammation. Under physiological conditions, these responses resolve once the epidermal barrier has been restored, allowing cutaneous homeostasis to be re-established (12, 120).

In atopic dermatitis (AD), genetic susceptibility, altered epidermal differentiation, inflammation, and scratching interact to impair the skin barrier. Human and murine studies implicate epithelial cytokines and cutaneous ILC2s, but the relative importance of TSLP, IL-25, IL-33, and other signals varies by model and tissue state (12, 121, 122). Persistent type 2 activation can amplify barrier dysfunction; it should not be assumed to represent only a prolonged version of an otherwise protective repair program.

Human AD lesions contain expanded ILC2 populations, and functional studies link skin ILC2s to epithelial cytokine responses (121, 122). Murine work also demonstrates a contribution of cutaneous ILC2s to inflammatory responses (120). Neuromedin U studies in lung and intestinal settings establish direct neuronal control of ILC2s, but they do not by themselves demonstrate that this pathway is central to human AD (14, 70, 123). Human single-cell and spatial studies instead reveal broader epithelial, stromal, and immune-cell programs in inflammatory skin disease; these tissue-level findings should not be interpreted as functional lineage tracing of cutaneous ILC2s (90, 124).

Neuroimmune signaling adds a further route by which type 2 inflammation can reinforce scratching and barrier injury. Sensory neurons respond to IL-4/IL-13-associated signaling through neuronal IL-4R*α* and downstream Janus kinase (JAK) pathways, linking inflammatory cytokines to chronic itch (125). This mechanism does not identify ILC2s as the exclusive cytokine source. Changes in epithelial differentiation and inflammatory cell organization likewise involve multiple populations (90, 124). The itch–scratch cycle therefore provides a disease-specific feedback mechanism within the broader failed-homeostasis framework.

Randomized trials of dupilumab and tralokinumab establish clinical benefit from targeting IL-4R*α* or IL-13 in AD, while trials of upadacitinib demonstrate benefit from broader JAK inhibition (126–128). These interventions support pathway relevance without isolating an ILC2-specific effect. Durable barrier recovery and control of itch should be evaluated alongside inflammatory improvement, and efficacy of downstream type 2 blockade should not be taken as evidence that epithelial alarmin blockade has equivalent clinical benefit.

### 4.6 Fibrosis and tissue remodeling: common consequences of persistent ILC activation

Although allergic diseases affect organs with distinct structural and physiological characteristics, they frequently converge on progressive tissue remodeling and loss of normal organ function. In the airways, skin, gastrointestinal tract, and sinonasal mucosa, recurrent epithelial damage prevents complete resolution of physiological repair responses and prolongs communication among tissue-resident ILCs, epithelial cells, fibroblasts, vascular cells, neurons, and infiltrating leukocytes. ILCs do not act as isolated effector populations within these tissues. They participate in multicellular networks that coordinate epithelial regeneration, stromal activation, and restoration of barrier function after injury. When these networks remain engaged for prolonged periods, however, the local environment is progressively reorganized, resulting in extracellular matrix accumulation, fibrosis, and impaired tissue function (12, 13, 44, 83, 88).

ILC2s can contribute to remodeling through several complementary pathways. IL-5 maintains eosinophilic inflammation, whereas IL-13 alters epithelial differentiation, stimulates mucus production, and promotes fibroblast and smooth-muscle responses. Activated ILC2s also produce amphiregulin and other mediators associated with epithelial regeneration and wound repair. These activities are beneficial after acute damage, when they help restore barrier integrity, but their prolonged expression can favor goblet-cell metaplasia, myofibroblast differentiation, collagen deposition, smooth-muscle hypertrophy, angiogenesis, and distortion of normal tissue architecture (4, 13, 60, 83). Experimental evidence also indicates that IL-33-responsive ILC2s accumulate near activated fibroblasts in fibrotic lung tissue and can directly enhance fibroblast collagen production, providing a mechanistic link between sustained ILC2 activation and matrix deposition (129).

Across organs, reduction in inflammatory biomarkers and reversal of established fibrosis are distinct outcomes. The evidence for ILC2-associated cytokine effects on remodeling is strongest in defined experimental settings, while human biopsy and clinical response data cannot generally assign structural change to one lymphocyte population. This distinction is especially important when interpreting a biologic response as support for a pathway rather than proof of an ILC2-specific remodeling mechanism (13, 130).

Fibroblasts are active participants in these responses rather than passive recipients of immune-derived signals. Single-cell studies have identified multiple fibroblast states with distinct anatomical distributions and functions in matrix production, immune-cell recruitment, and epithelial support (87, 88). Reciprocal signaling among fibroblasts, epithelial cells, macrophages, eosinophils, mast cells, endothelial cells, neurons, and ILCs can therefore establish local niches in which inflammation and repair reinforce one another. Spatial profiling of fibrotic lung tissue has similarly revealed organized pathological neighborhoods containing myofibroblasts, abnormal epithelial populations, and immune cells, illustrating how remodeling is maintained through cellular organization within the tissue rather than through a single cytokine pathway (131). Persistent epithelial stress can stabilize these interactions even after the intensity of the original environmental exposure has declined.

Changes in the extracellular matrix further influence this process. Increasing matrix stiffness alters fibroblast differentiation, epithelial behavior, and immune-cell function, creating feedback loops in which the remodeled tissue itself promotes additional remodeling. In a silicosis model, experimental work has shown that mechanically activated fibroblasts can alter local ILC states and influence the progression of silica-associated pulmonary fibrosis, supporting a bidirectional relationship between the physical properties of the tissue and resident immune populations (62). Although such mechanisms have been studied most directly in fibrotic lung models, they provide a broader explanation for how structural alterations may become partly self-maintaining once extensive matrix remodeling has occurred.

Reduction in inflammatory activity and reversal of structural abnormalities are distinct outcomes. This is particularly relevant to fibrostenotic EoE, while remodeling in asthma, CRSwNP, and AD differs in anatomy and clinical significance (13, 89). Trials demonstrating improved symptoms, exacerbations, or polyp scores establish therapeutic benefit but do not automatically demonstrate complete restoration of tissue architecture (107, 113, 119). Longitudinal structural measures are needed to test whether inflammatory control also alters the course of remodeling.

Evidence across allergic diseases supports a model in which recurrent environmental injury repeatedly engages physiological repair pathways involving tissue-resident ILCs. Failure to terminate these responses converts normally protective communication among immune and structural cells into prolonged inflammatory and remodeling circuits. The resulting pathology reflects neither ILC activity alone nor an intrinsically abnormal repair mechanism, but the duration and tissue context in which these programs operate. Defining the signals that permit successful resolution—and those that stabilize fibroblast activation, altered epithelial differentiation, and pathological matrix deposition—may reveal strategies that prevent disease progression while preserving the essential functions of ILCs in barrier maintenance and tissue repair (1, 2, 13, 88).

## 5 Restoring tissue homeostasis: future perspectives

The evidence reviewed here supports a tissue-contextual view of ILC function: developmental programs establish lineage potential, while local niches influence mature responses (3, 11, 12). This does not establish that every ILC subset has the same causal contribution in every organ. In allergic disease, ILC2s are the principal focus, whereas ILC1-, ILC3-, and intermediate-state contributions depend on phenotype and experimental context (32, 45).

We propose failed restoration of tissue homeostasis as a conceptual framework, not a single mechanism shared uniformly across allergic diseases. Repeated epithelial injury can sustain alarmin-associated type 2 circuits, but the initiating exposures, contribution of adaptive immunity, tissue architecture, and remodeling responses differ among asthma, CRSwNP, EoE, and atopic dermatitis. Murine studies establish mechanistic possibilities, whereas patient tissue and clinical interventions test distinct aspects of relevance; neither alone proves that ILC2s drive every clinical phenotype (3, 4, 35).

Single-cell and chromatin-accessibility analyses reveal heterogeneity among human ILC populations, while spatial studies add anatomical relationships that dissociated profiles cannot capture (34, 38, 39). These approaches remain largely observational in human tissue. Their integration with perturbation, longitudinal sampling, and appropriately matched animal or *ex vivo* models will be needed to test whether a proposed niche signal actually controls ILC behavior rather than merely accompanying inflammation.

Structural cells can shape ILC niches, but the direction and clinical relevance of stromal–ILC communication depend on the injury context. A pro-resolving CD200-positive fibroblast network was identified in arthritis, whereas mechanically activated fibroblasts promoted ILC2 plasticity in experimental silicosis (61, 62). These contrasting outcomes illustrate the potential for stromal signals to support resolution or persistent pathology; neither study alone establishes the corresponding mechanism in allergic airway or skin disease.

Therapeutic evidence must be interpreted at the level actually tested. Clinical responses to blockade of TSLP, IL-4R*α*, or eosinophil-associated pathways support the importance of these pathways in defined disease populations, but the agents act on multiple cell types and do not demonstrate ILC2-specific causality. IL-33/ST2 and neuroimmune interventions require separate assessment according to their clinical or experimental evidence. Table 1 distinguishes intervention, disease setting, evidence level, and limitations.

**Table 1:** Therapeutic pathways relevant to ILC2-associated allergic inflammation: disease context, evidence, and limitations.

**Target and intervention**	**Disease context**	**Evidence**	**Limitations**
TSLP: tezepelumab	Severe asthma; CRSwNP	Human phase 3 trials (108, 116).	Shared upstream pathway; benefit does not isolate ILC2 effects or establish reversal of remodeling.
IL-4R*α*: dupilumab	Asthma; CRSwNP; EoE; AD	Human randomized trials (107, 113, 119, 126).	Multiple responsive cell types; tissue repair and structural outcomes require separate assessment.
IL-5: mepolizumab; IL-5Rα: benralizumab	Severe eosinophilic asthma; mepolizumab studied in CRSwNP	Human phase 3 trials (105, 106, 115).	Predominantly eosinophil-associated effects; not selective ILC2 inhibition; indications differ by agent.
IgE: omalizumab	Allergic asthma; CRSwNP	Human phase 3 CRSwNP trials (114).	IgE-pathway benefit does not establish an ILC2-specific mechanism; phenotype and eligibility matter.
IL-13: tralokinumab	Moderate-to-severe AD	Human phase 3 trials (127).	Downstream cytokine blockade across cell sources; results cannot be generalized to all allergic diseases.
JAK inhibition: upadacitinib	Moderate-to-severe AD	Human phase 3 trials (128).	Broad intracellular effects; infection and other safety risks require disease-specific clinical assessment.
IL-33 blockade: itepekimab; ST2 pathway	Asthma; other barrier settings under investigation	Human phase 2 asthma trial for itepekimab (109).	Trial evidence is agent- and disease-specific; IL-33 also participates in repair and regulation; not an established selective ILC2 therapy.
NMU and *β*2-adrenergic signaling	Experimental intestinal or pulmonary type 2 responses	Predominantly murine mechanistic studies (14, 41, 70).	Neural pathways have effects beyond ILC2s; preserving repair while limiting inflammation requires direct testing.
Fatty-acid metabolism	Malnutrition and helminth-associated barrier injury	Murine mechanistic evidence (43).	Experimental context differs from allergy; inhibiting a metabolic requirement could also impair protective responses.

Therapeutic suppression presents a double-edged problem: ILC2-associated pathways can amplify type 2 inflammation yet also participate in epithelial repair in experimental injury models. Broad blockade might affect protective responses, but its magnitude and clinical relevance cannot be inferred directly from mouse knockout studies. The appropriate comparison is disease-specific benefit against measured effects on infection, barrier recovery, and long-term remodeling; neuroimmune and metabolic approaches remain experimental.

The main unresolved issues are causal rather than merely descriptive: which ILC states persist after injury, which spatially inferred interactions are functional, and which tissue-repair activities can be preserved during anti-inflammatory treatment? Human longitudinal tissue studies, perturbable *ex vivo* systems, and matched mouse models will be complementary rather than interchangeable sources of evidence.

In conclusion, ILC biology has evolved from the study of a previously unrecognized lymphocyte population to a broader understanding of how barrier tissues preserve physiological homeostasis. The evidence reviewed here supports a model in which ILCs participate in multicellular tissue networks that integrate immune and structural signals to maintain tissue integrity throughout life. When epithelial injury is transient, these interactions promote repair and successful resolution. When environmental injury becomes persistent, however, protective responses may become prolonged and contribute to chronic inflammation, tissue remodeling, and organ dysfunction. Understanding how these multicellular regulatory networks are established, maintained, and restored is therefore likely to define the next generation of ILC research and may ultimately provide new opportunities to prevent and treat allergic diseases by restoring tissue homeostasis rather than simply suppressing inflammation.
